# Dispersion Curve Engineering of TiO_2_/Silver Hybrid Substrates for Enhanced Surface Plasmon Resonance Detection

**DOI:** 10.3390/s16091442

**Published:** 2016-09-07

**Authors:** Sherif H. El-Gohary, Munsik Choi, Young L. Kim, Kyung Min Byun

**Affiliations:** 1 Department of Biomedical Engineering, Kyung Hee University, Yongin 17104, Korea; Sherif.hamdy@khu.ac.kr (S.H.E.-G.); blue-sky031@hanmail.net (M.C.); 2 Department of Computer Science and Engineering, Kyung Hee University, Yongin 17104, Korea; 3 Weldon School of Biomedical Engineering, Purdue University, West Lafayette, IN 47907, USA

**Keywords:** surface plasmon resonance biosensor, dispersion engineering, sensitivity, stability

## Abstract

As surface plasmon resonance (SPR)-based biosensors are well translated into biological, chemical, environmental, and clinical fields, it is critical to further realize stable and sustainable systems, avoiding oxidation susceptibility of metal films—in particular, silver substrates. We report an enhanced SPR detection performance by incorporating a TiO_2_ layer on top of a thin silver film. A uniform TiO_2_ film fabricated by electron beam evaporation at room temperature is an effective alternative in bypassing oxidation of a silver film. Based on our finding that the sensor sensitivity is strongly correlated with the slope of dispersion curves, SPR sensing results obtained by parylene film deposition shows that TiO_2_/silver hybrid substrates provide notable sensitivity improvement compared to a conventional bare silver film, which confirms the possibility of engineering the dispersion characteristic according to the incidence wavelength. The reported SPR structures with TiO_2_ films enhance the sensitivity significantly in water and air environments and its overall qualitative trend in sensitivity improvement is consistent with numerical simulations. Thus, we expect that our approach can extend the applicability of TiO_2_-mediated SPR biosensors to highly sensitive detection for biomolecular binding events of low concentrations, while serving a practical and reliable biosensing platform.

## 1. Introduction

Optical detection technologies based on surface plasmon resonance (SPR) are commonly used in a variety of biological, chemical, environmental, and clinical applications because they are very powerful tools for monitoring binding interactions in a label-free manner [1,2,3,4,5]. When wave-vectors of a polarized incidence beam and surface plasmons are equal, the phenomenon of SPR takes place and the reflected light intensity becomes completely attenuated. Since the SPR signal is sensitive to variations in the refractive index or the thickness of a sensing medium on top of a metallic substrate, one can measure the adsorption of target analytes by tracking changes under the resonance condition [6,7,8]. Typically, a metal film to excite the surface plasmons includes gold and silver [9,10]. Gold has been the most widely used as it has stable optical and chemical properties. Although silver can provide a sharper SPR curve as an attractive substrate, the major disadvantage of chemical instability makes it difficult to obtain reliable optical signals and to perform long-time measurements, limiting the practicality of silver-based SPR biosensors [11].

In this respect, several approaches have been suggested to prevent a silver film in SPR biosensors from being oxidized when it is exposed to an oxygen-containing ambience as the commonly biosensing environment. In order to make the best use of silver-based SPR interfaces, a thin protecting layer should be employed to stabilize the interface while maintaining favorable advantages of silver in terms of SPR signal quality. For example, the introduction of a gold overlayer was proposed to increase the sensor sensitivity as well as to block oxidation [12,13]. Choi et al. presented a numerical analysis for verifying the possibility of utilizing a graphene-silver film for highly sensitive SPR imaging detection [14]. Moreover, good electrical and electrochemical characteristics were accomplished by combining a conducting oxide layer such as indium tin oxide (ITO) with a silver film [15,16]. Sensor sensitivity in SPR detection could also be improved by using a high refractive index dielectric layer such as Si and AlAs on top of a metal film [17,18,19]. 

However, the use of absorptive materials such as metal or graphene inevitably produces a degradation in SPR signal quality due to a high imaginary part in the refractive index. Reflectance with shallow depth and broad bandwidth at resonances increases the uncertainty of the sensor output and generates significant errors in the experimental measurements, resulting in notable deterioration of the detection limit. On the other hand, a non-absorbing dielectric film with a low refractive index requires a long process-time to produce micrometer-scale thickness for sensitivity enhancement. Time-consuming and labor-intensive fabrication in a chamber at high temperature may cause a severe damage or degeneration of the silver surface. In addition, a dielectric overlayer with a refractive index larger than three in visible bands, its optimal thickness for sensitivity enhancement was determined to be around or less than 10 nm, which inevitably demands a great accuracy in actual thin-film fabrication to guarantee a reliable sensing performance [17,18,19].

In this study, we propose titanium oxide (TiO_2_) of a fairly high refractive index [20] as an effective dielectric material for enhancing the sensor sensitivity as well as a process-compatible protection layer for preventing a silver substrate from oxidation. Based on our recent finding that the sensitivity of SPR biosensor is strongly correlated with its dispersion curve characteristics [21], we show that a TiO_2_ overlayer is advantageous for engineering the dispersion relation of a silver film. In water and air ambiences, the silver-based SPR biosensor combined with a TiO_2_ film can be optimized to produce an enhanced sensitivity with respect to a traditional silver film. We further discuss the sensitivity improvement by analyzing the near-field distribution and field-matter interactions in the proximity of the TiO_2_/silver hybrid substrate.

## 2. Numerical Methods

For performance analysis of a multi-layered SPR configuration, we utilize the transfer-matrix method (TMM). In our TMM computation, the reflectance is calculated based on a 2 × 2 matrix, which is a serial product of the interface matrix *I_jk_* (*j* = 0, 1, 2, 3, and *k* = *j* + 1) and the layer matrix *L_j_* as follows:

(1) $$R={\left|\frac{M_{12}}{M_{22}}\right|}^{2},$$

where

(2) $$M=\left[\begin{array}{cc} M_{11} & M_{12}\\ M_{21} & M_{22} \end{array}\right]=I_{01}L_{1}I_{12}L_{2}I_{23}L_{3}I_{34},$$

(3) $$I_{jk}=\left[\begin{array}{cc} 1 & r_{jk}\\ r_{jk} & 1 \end{array}\right],\text{ and }L_{j}=\left[\begin{array}{cc} e^{ik_{zj}d_{j}} & 0\\ 0 & e^{-ik_{zj}d_{j}} \end{array}\right].$$

Here, *r_jk_*, *k_zj_*, and *d_j_* represent the Fresnel reflection coefficient, wave vector in the *z*-direction, and the thickness of *j*th layer, respectively. *r_jk_* and *k_zj_* are given by:

(4) $$r_{jk}=\frac{\left(\frac{k_{zj}}{\varepsilon_{j}}-\frac{k_{zk}}{\varepsilon_{k}}\right)}{\left(\frac{k_{zj}}{\varepsilon_{j}}+\frac{k_{zk}}{\varepsilon_{k}}\right)}\text{ and}$$

(5) $$k_{zj}=\sqrt{\varepsilon_{j}{\left(\frac{\omega }{c}\right)}^{2}-k_{x}^{2}}\text{ with }k_{x}=\sqrt{\varepsilon_{0}}\frac{\omega }{c}\sin\theta ,$$

where *ω* is the angular frequency, *c* is the speed of light in free-space, and *ε*_0_ is the optical constant of a prism substrate. The details of the TMM algorithm can be found elsewhere [22]. Both wavelength scanning from 380 to 1500 nm with an increment of 10 nm and angle scanning from 30° to 80° with an increment of 0.01° are performed to obtain reflectance curves and dispersion characteristics.

**Figure 1:**
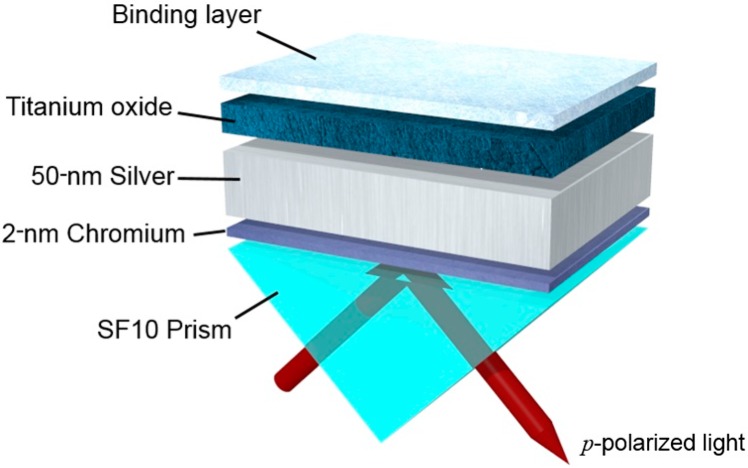
Schematic of the proposed surface plasmon resonance (SPR) structure with TiO_2_/silver hybrid substrate. A 50-nm-thick silver film is deposited on SF10 prism via a chromium layer with a 2-nm thickness. TiO_2_ layer is deposited between silver film and binding layer. A *p*-polarized light is incident through the prism substrate with an illumination angle of *θ*.

A schematic diagram of the proposed SPR substrate is presented in Figure 1. A thin silver film with a thickness of 50 nm and a dielectric TiO_2_ layer is deposited onto SF10 glass prism via an adhesion of a 2-nm thick chromium layer. It should be emphasized that, since the resonance is found at a high angular momentum due to the presence of a TiO_2_ layer, we choose the prism substrate as SF10 with a high refractive index to satisfy the phase-matching condition at a smaller incidence angle [23]. The optical constants and dispersion values for thin films of silver, SF10, and TiO_2_ are referred from the published data [24,25].

## 3. Experimental Details

In order to verify if a TiO_2_ layer is a viable option for protecting a silver film from oxidation and improving SPR detection, we fabricate a hybrid SPR substrate with a varied TiO_2_ thickness using thin film formation processes such as electron beam evaporation (UEE, ULTECH, Daegu, Korea). By means of reactive gas flow between evaporating titanium electrodes that are heated by the electron beam, thin and transparent TiO_2_ coatings could be produced in the chamber is 5 × 10^−6^ torr at room temperature, realizing a slow deposition rate of 0.2 nm/s [26]. After the deposition, the fabricated samples are cleaned in 70% ethanol solution for 10 min in a sonication bath and rinsed with distilled deionized-water to remove any residues.

We perform characterizations of 50-nm-thick silver samples with and without TiO_2_ layer, with an in-house optical setup using an intensity-based angular interrogation scheme. Our setup employs a polarized He-Ne laser of *λ* = 633 nm and dual rotation stages (URS75PP, Newport, Irvine, CA, USA), pre-aligned for the sensor chip and a calibrated photodiode (818-UV, Newport, Irvine, CA, USA), with a nominal resolution of 0.002°. During the experiments, SPR curves are measured with a resolution of 0.01°. The sensor sensitivities of silver samples with and without TiO_2_ coating are determined by comparing the resonance angles before and after parylene film deposition. Although the sensitivity obtained from layered bio-reactions such as antigen-antibody interaction and DNA hybridization would be a more practical performance measure, the degree of biomolecular interactions cannot be identical for metal and dielectric surfaces and the immobilization efficiency of the ligands varies with the substrate material. On the other hand, a parylene film with a good adhesion regardless of the substrate material has been utilized as a linker layer to promote the covalent immobilization of proteins in SPR biosensing applications [27,28]. Note that a thickness of the parylene film is determined as less than 10 nm to induce an effective field-matter interaction within the penetration depth of surface plasmon fields [29].

## 4. Results and Discussion

**Figure 2:**
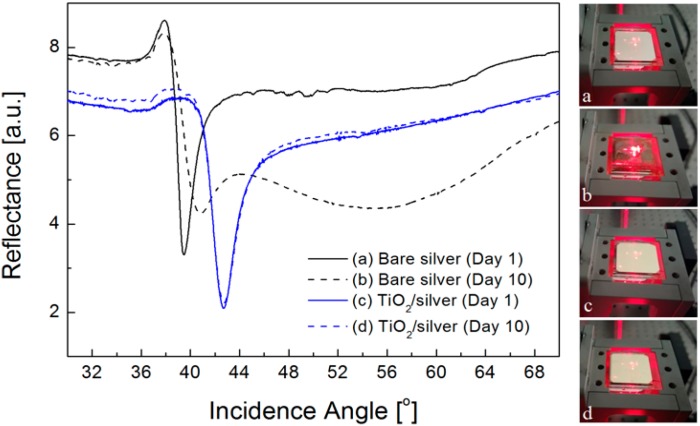
Experimental reflectance curves and photo images for SPR samples with and without 30-nm thick TiO_2_ overlayer. Solid and dotted lines indicate the samples at the 1st and 10th day after exposure of bare silver (black lines) and TiO_2_/silver (blue lines) substrates to the air environment, respectively. (**a**) SPR sample with bare silver film at day 1; (**b**) SPR sample with bare silver film at day 10; (**c**) SPR sample with TiO_2_/silver film at day 1; (**d**) SPR sample with TiO_2_/silver film at day 10.

First of all, in order to confirm that the presence of TiO_2_ prevents a silver film from being oxidized, we measure the SPR curves of silver substrates with and without a 30-nm thick TiO_2_ layer. It has been known that silver oxidation occurs at the expense of the underlying silver film and the converted silver layer significantly alters its optical properties, leading to changes in the SPR characteristics [30,31]. As illustrated in Figure 2, exposure of a silver film with a TiO_2_ layer to an oxygen gas for 10 days presents no change in SPR curves, while a bare silver film exhibits a notable shift and deformation of resonance band, which is associated with a transition of optical constants from silver to silver oxide. Obviously, the introduction of TiO_2_ can protect the optical property of a silver surface and thus preserve its plasmonic behavior.

**Figure 3:**
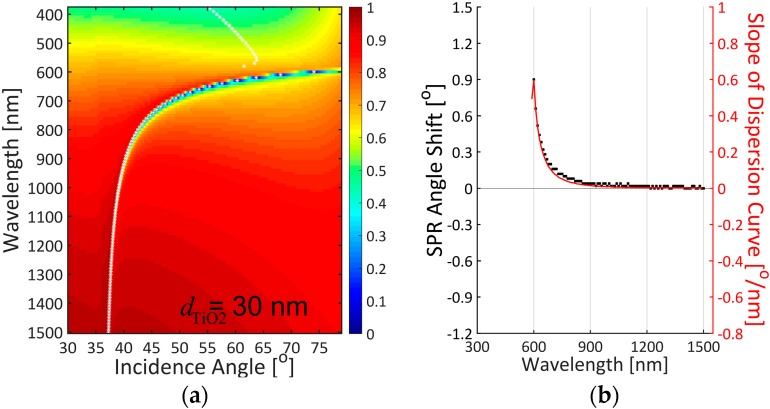
(**a**) 2D dispersion curve; (**b**) The relation between slope of dispersion curve and SPR angle shift for the silver substrates with 30-nm thick TiO_2_ film. Video S1 shows the calculation results when the thickness of TiO_2_ layer varies from 0 to 70 nm with a step of 5 nm.

From the TMM calculation results in Figure 3, the slope of dispersion curves (solid line in red), i.e., Δ*θ*_SPR_/Δ*λ*, is greatly correlated with SPR angle shift (squares in black) for the silver film with a 30-nm thick TiO_2_ layer. In our TMM calculation, SPR angle shifts are determined by finding a difference in resonance angles when the refractive index of 5-nm thick binding layer changes from 1.40 to 1.45. Note that SPR curves with minimum reflectance larger than 0.2 are discarded in determining the SPR angle, since a shallow resonance dip is not appropriate for precise and accurate detection practically. Interestingly, manipulation of dispersion curve by an added SiO_2_ overlayer on top of a silver film was utilized to expand the color dispersion of SPR-based holography to incident angle because the angular separation for color reconstruction is relatively small for the case without SiO_2_ layer [32]. Inspired by the previous work, we find a possible link between dispersion relation and SPR sensor sensitivity. The strong correlation between the slope of dispersion curve and the sensor sensitivity is attributed to the fact that dispersion relation in dielectric-metal interface relates the angular frequency of surface plasmon field to its wave-vector magnitude depending on the optical constants of dielectric and metallic materials. The dispersion curve is thus slightly displaced due to a refractive index change at the binding region. This gives us an interesting postulation that the slope of dispersion curve can be used not only to predict wavelength-dependent sensitivity characteristics for a given SPR scheme but also to optimize a maximally obtainable sensitivity with no need of calculating all the resonance angles and their shifts before and after a binding event.

**Figure 4:**
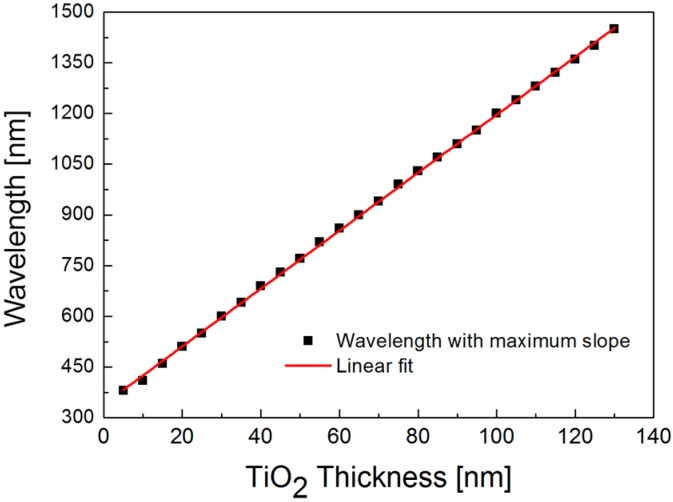
Linear relation between incidence wavelength and TiO_2_ thickness with a peak sensor sensitivity.

In the video linked to Figure 3, when a TiO_2_ thickness varies from 0 to 70 nm with a step of 5 nm in an air ambience, we confirm the correlation between the slope of dispersion curve and the sensor sensitivity. Especially, as the distribution of reflectance minimum in a dispersion curve bends toward a longer wavelength and a higher incidence angle, SPR excitation in the visible range is not found for the thickness of TiO_2_ larger than 40 nm. It is also observed that the bended dispersion curve leads to a gradual decrease in the maximum slope due to a reduced contrast in wavelength. More interestingly, a highly linear relation between the optimal wavelength in sensitivity and the TiO_2_ thickness is obtained by engineering the dispersion curve as shown in Figure 4. This linearity can facilitate designing an optimized SPR structure by choosing a proper TiO_2_ thickness for a given incidence wavelength. As a result, dispersion curve engineering is useful in optimizing the wavelength-dependent sensor sensitivity.

**Figure 5:**
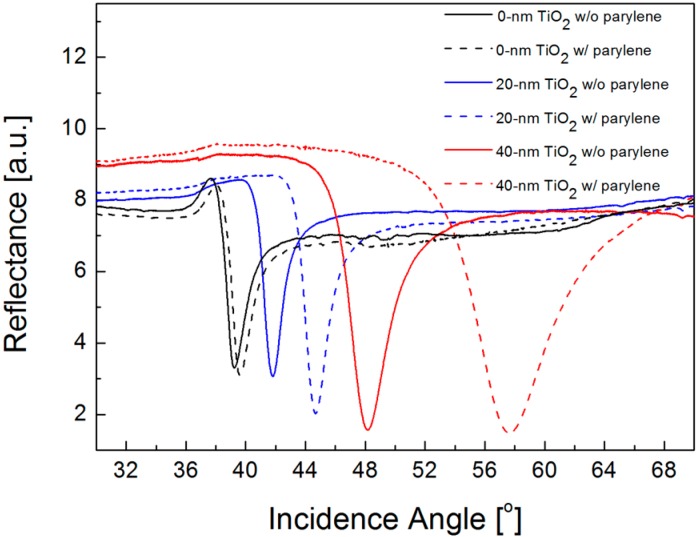
Experimental reflectance curves versus incidence angle for bare silver samples without TiO_2_ film (black lines) for silver films with 20-nm (blue lines) and 40-nm thick TiO_2_ film (red lines). All the curves in solid and dotted lines indicate the measurement data before and after the deposition of 10 nm thick parylene film in the air environment.

Since SPR biosensors often suffer from modest sensitivity in detecting a biomolecular interaction of low molecular weights or low concentrations, the dispersion curve of TiO_2_/silver hybrid substrate needs to be engineered to enhance the sensitivity by changing the thickness of TiO_2_ film. For the wavelength of *λ* = 633 nm, one of the most commonly used spectral lines in a practical SPR biosensor, an optimal TiO_2_ thickness in an air ambience lies between 30 and 40 nm from the linear fit in Figure 4. Hence, we fabricate the SPR samples of a silver film without TiO_2_ layer and 20- and 40-nm thick TiO_2_/silver hybrid substrates and compare the sensor sensitivity by measuring SPR curves before and after depositing a 10-nm thick parylene film. In Figure 5, for a bare silver film, resonance angle shifts from 39.23° to 39.60°; thus, the net change is 0.37°. On the other hand, the measured SPR angle shifts are, respectively, 2.88° and 9.49° for 20- and 40-nm thick TiO_2_ layers, which corresponds to 7.8-fold and 25.7-fold sensitivity enhancements. While this enhancement is not an accurate estimation due to a large refractive index change after parylene film deposition, the qualitative trends of increased sensitivity with a thicker TiO_2_ layer before reaching an optimal thickness resemble the results obtained by the correlation study in Figure 3.

**Figure 6:**
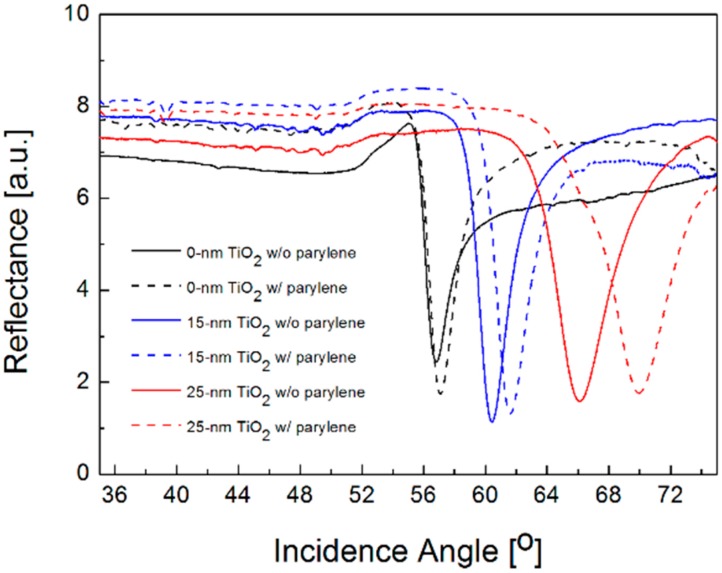
Experimental reflectance curves versus incidence angle for bare silver samples without TiO_2_ film (black lines) for silver films with 15-nm (blue lines) and 25-nm thick TiO_2_ film (red lines). All the curves in solid and dotted lines indicate the measurement data before and after the deposition of 5 nm thick parylene film in water solution.

While not shown here, it is also confirmed in water ambience that the slope of dispersion curve is highly correlated with the sensor sensitivity and the relation between the optimal thickness of TiO_2_ layer and the operating wavelength is linear. The optimum TiO_2_ thickness in water solutions is determined to be 25 nm for the wavelength of *λ* = 633 nm, which is smaller than the value in air environment because the resonance angle moves to a higher momentum. From parylene coating with a thickness of 5 nm, Figure 6 shows the measured sensitivity when the thickness of TiO_2_ layer is 0, 15, and 25 nm. Compared to the resonance shift of 0.26° for bare silver film, the average SPR angle shifts are 1.20° and 3.87°, and thus the enhancement is obtained as 4.7 and 14.9 times for TiO_2_ thickness of 15 and 25 nm, respectively. Although actual sensitivity improvement obtained by a small refractive index change on a sensor surface would be much less than the enhancement by parylene film deposition, such significant improvement verifies the possibility of engineering the dispersion curve according to TiO_2_ thickness and selecting its optimal thickness to enhance the performance of SPR detection by making the best of a correlation between the slope of dispersion curve and the sensitivity for a given SPR structure.

**Figure 7:**
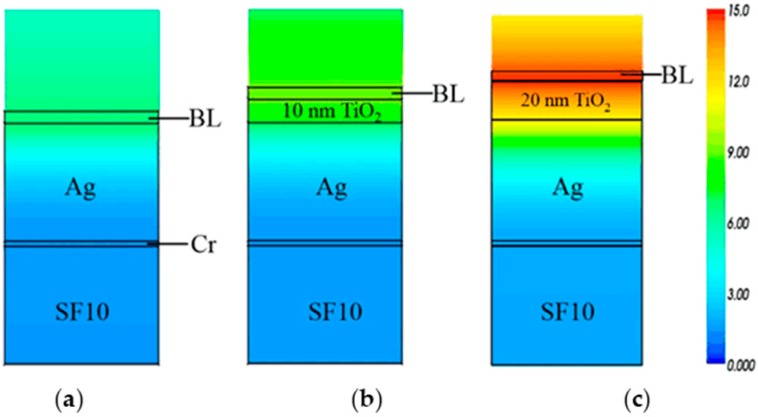
Finite-difference time-domain (FDTD) results of SPR substrates with (**a**) a bare silver film; (**b**) 10 nm; and (**c**) 20 nm thick TiO_2_ film on top of silver film. Two-dimensional FDTD images are normalized by the field amplitude of 15.

Furthermore, we investigate an effect of TiO_2_ thickness on plasmon field amplitude. This is based on the postulation that field-matter interaction plays an important role in determining the sensor sensitivity. From the previous studies [21,33], the field amplitude at the binding region is greatly correlated with the sensitivity, and, hence, as a quantitative metric of field-matter interaction, near-field characteristics can be a useful tool to assess the performance of SPR biosensors that address an enhancement of detection sensitivity. For conventional and proposed SPR structures in water ambience, implying a practical biosensing condition, the finite-difference time-domain (FDTD) results in Figure 7 visualizing the distributions of near-field amplitude for E_X_ component at the wavelength of *λ* = 630 nm near the sensor surface. Based on the assumption that the field of an incident beam is of unit amplitude, the maximum E_X_ field is found at the interface between the substrate and the binding layer and the plasmon field amplitude is decayed exponentially along the distance from a substrate surface. The peak amplitude is obtained as 6.58 for bare silver film and 8.45 and 13.95 for 10 and 20 nm thick TiO_2_ layers. 

**Figure 8:**
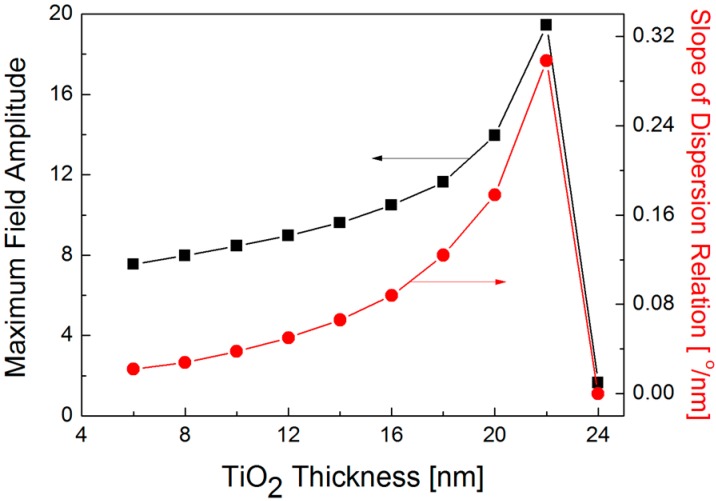
Correlation between maximum field amplitude (black squares) and slope of dispersion relation (red circles) when TiO_2_ thickness varies from 6 to 24 nm with a step of 2 nm at the wavelength of *λ* = 630 nm.

Figure 8 shows that both peak value of field amplitude and slope of dispersion curve reach a maximum at the TiO_2_ thickness of 22 nm for an incidence wavelength of *λ* = 630 nm. When a thickness of TiO_2_ layer increases, the overall trend in the peak amplitude of E_X_ field matches the profile of the slope of dispersion curve. Although an optimal TiO_2_ thickness should be chosen as 22 nm to accomplish the highest sensitivity, it seems to be more appropriate to select a suboptimal of TiO_2_ thickness at around 20 nm because an abrupt decrease in the peak amplitude and the slope of dispersion curve is found when TiO_2_ thickness is larger than 22 nm and any fabrication error during TiO_2_ deposition may degrade the sensitivity. On the other hand, if it is not possible to precisely control the TiO_2_ thickness, it would be better to change the wavelength of the light source to an optimal value for a given TiO_2_ thickness according to the linear relation in Figure 4.

One undesirable effect of TiO_2_ in the SPR configuration is semiconductor photocatalysis, which can be activated by plasmon-induced electron inject from silver to TiO_2_. Hot electrons from the silver layer via the intra-band transitions in silver under the SPR resonance could migrate to TiO_2_, when their energy is large than the Schottky barrier between the metal and the semiconductor [34]. Thus, we also test photocatalysis experiments using methylene blue, in which any photocatalysis effects can result in degradation of methylene blue. While the results are not presented, any notable change in the resonance angle and the color of methylene blue is not found during one-hour exposure of TiO_2_/silver substrate to a laser light of *λ* = 633 nm at its resonance condition. Our negative results indicate that, in the proposed SPR configuration, electrons and holes may be recombined during the transfer process to the surface of the TiO_2_. These inefficient oxidation-reduction reactions can be additional benefits for using TiO_2_ for biosensing applications.

## 5. Conclusions

In this study, we demonstrate that the addition of a TiO_2_ layer with a high refractive index on top of a silver film is advantageous for protection of the silver surface and improvement in the SPR biosensor sensitivity. As we find that the sensor sensitivity is correlated with the slope of dispersion curve, engineering the dispersion curve by varying the TiO_2_ thickness can be an effective means in obtaining the optimal performance in sensitivity for a given wavelength. In both air and water ambiences, the experimental results show that the selection of optimal or suboptimal thickness of TiO_2_ layers could provide a notable enhancement in sensitivity, compared to the case of bare silver film. Based on the principle of field-matter interaction, we show that the improved detection sensitivity is consistent with the peak plasmon field amplitude at the sensor surface. Thus, we expect that the proposed TiO_2_/silver substrate and its engineered dispersion curve could be promising to realize a high-sensitivity SPR biosensor for application in a variety of biomolecular reactions.

## Supplementary Materials

The following are available online at http://www.mdpi.com/1424-8220/16/9/1442/s1. Video S1: correlation between dispersion curve slope and SPR angle shift for a varied TiO_2_ thickness.
